# Determinants of family planning use among married women in bale eco-region, Southeast Ethiopia: a community based study

**DOI:** 10.1186/s12905-018-0539-7

**Published:** 2018-03-12

**Authors:** Alemayehu Gonie, Alemayehu Wudneh, Dejene Nigatu, Zelalem Dendir

**Affiliations:** 1 Department of Nursing, School of Health Science, Goba Referral Hospital, Madda Walabu University, Bale-Goba, Ethiopia; 2 Department of Natural Resource Management, College of Agriculture and Natural Resources, Madda Walabu University, Bale-Robe, Ethiopia

**Keywords:** Contraceptive, Married women, Bale eco-region

## Abstract

**Background:**

Family planning is the ability of individuals and couples to anticipate and attain their desired number of children and the spacing and timing of their births. Providing family planning could prevent maternal deaths by allowing women to delay motherhood, space births, avoid unintended pregnancies and abortions, and stop childbearing when they reach their desired family size. Despite the fact that family planning is advantageous for maternal and newborn health and the services and commodities are free of charge, the reason of not using modern family planning methods is unclear in Bale Eco-Region. Therefore, this study assessed the contraceptive prevalence rate and its determinants among women in Bale Eco-Region, Ethiopia.

**Methods:**

A community-based cross-sectional study design (both quantitative and qualitative methods) was conducted from December 2016 to February 2017. Five hundred sixty-seven women were successfully interviewed using structured and pre-tested questionnaire. A multistage sampling technique was employed. Data were entered into Epi-data version 3.1 and exported to SPSS version 21. Logistic regression analyses were done and a significant association was declared at *p*-value less than 0.05. All focus group discussions and key informant interviews were recorded and analyzed thematically.

**Results:**

The overall contraceptive prevalence rate was 41.5%. Injectable (48.1%), implants (22.6%) and pills (20.0%) were the most contraceptive methods utilized by study participants. Spousal (husband’s) opposition (38.8%), religious beliefs (17.7%), concern and fear of side effects (14.8%), and distance of family planning service (5.9%) were the reasons for not using contraceptive methods. Having more than seven deliveries (AOR = 2.98, CI = 1.91–6.10, *P* = 0.000) and having birth interval less than 24 months between the last two children (AOR = 3.8, CI = 13.41–21.61, *P* = 0.003) were significantly associated with utilization of contraceptive methods.

**Conclusion:**

Low contraceptive prevalence rate might be attributed by husband opposition, religious beliefs, concern and fear of side effects. Having more than seven deliveries and birth interval less than 24 months between the last two children were determinants of contraceptive use. Family planning consultation opportunities should be created to make male’s involved and to increase their responsibility for family planning use.

## Background

Family planning (FP) is the ability of individuals and couples to anticipate and attain their desired number of children and the spacing and timing of their births. It is achieved through the use of contraceptive methods [1]. Family planning has public health, economic, and environmental importance and lessens stress on the natural resources and political environments at the national level [2]. It is the most cost-effective health and development investments available to governments [3]. It brings transformational benefits to women, families, communities, and countries, and helps females in achieving an educational goal, start a business plan, and achieve their employment needs [4, 5]. It improves the health of women and children by reducing the risk of unsafe abortion, low birth weight, preterm birth, and premature rupture of membranes [4, 6].

Even though studies had been conducted on barriers to family planning use, results were either consistent or contrasting. In Tanzanian study, FP counseling given at health care facilities was predictors of modern contraceptive use [7]. Aqeela T et al. [8] found that dissatisfaction with contraceptive methods, bad experiences with side effects, and privacy concerns were strongly affecting modern contraceptive use. Similarly, findings from Kenya and Nigeria, concern and fear of side effects were the major reasons for not using modern contraceptives [9, 10].

According to integrated family health program report in Ethiopia, discussing with husband about family planning use and knowing health extension workers are providing family planning services were significantly associated with modern family planning use [11]. In pastoralist community of Afar region, the attitude of women towards family planning methods, monthly income, and educational status increased modern contraception utilization [12]. In Gedeo zone, Ethiopia, residence, educational level, number of live children, women’s desire to have more children, women’s knowledge and attitude towards contraceptive methods were associated with FP utilization [13].

Currently, in Ethiopia, family planning services are integrated into maternal and child health care services at all levels of the health care delivery system and the service has been provided to the rural communities at the health posts through health extension workers. Considerable efforts have been made by the government, local and international partners on FP programs [14] and the commodities are free of charges. Despite all these efforts, the national modern contraceptive prevalence rate and unmet need for FP were reported as 35% and 22% in Ethiopian demographic health survey 2016, respectively which was lower than the global FP targets [15]. In addition, the prevalence of FP utilization was varied in different parts of the community; Hadiya zone (23.9%), South Gondar zone (66.2%), Gedeo zone (64.2%), Illubabor zone (44.9%), pastoralist community of Afar region (8.5%), pastoralist women in Bale zone (20.8%) [12, 13, 16–19].

In Bale Eco-region, there is a preconception or notion that having numerous children is regarded as beneficial. This suggests that the root cause of not using modern family planning methods is unclear. Hence, this study assessed contraceptive prevalence rate and its determinants among married women in Bale Eco-region, Ethiopia.

## Methods

### Study area and period

A community-based cross-sectional study (both quantitative and qualitative methods) was conducted from December 2016 to February 2017 in Bale Eco-region, Southeast-Ethiopia. The Eco-region has 16 districts and classified into three Agro-ecologies namely; highland (Gololcha, Gasera, Sinana, Dinsho), midland (Goro, Goba, Agarfa,) and lowland (Harena-Buluk, Dello-Mena, Madda-walabu and Gura-Damole, Berbere).

**Fig. 1:**
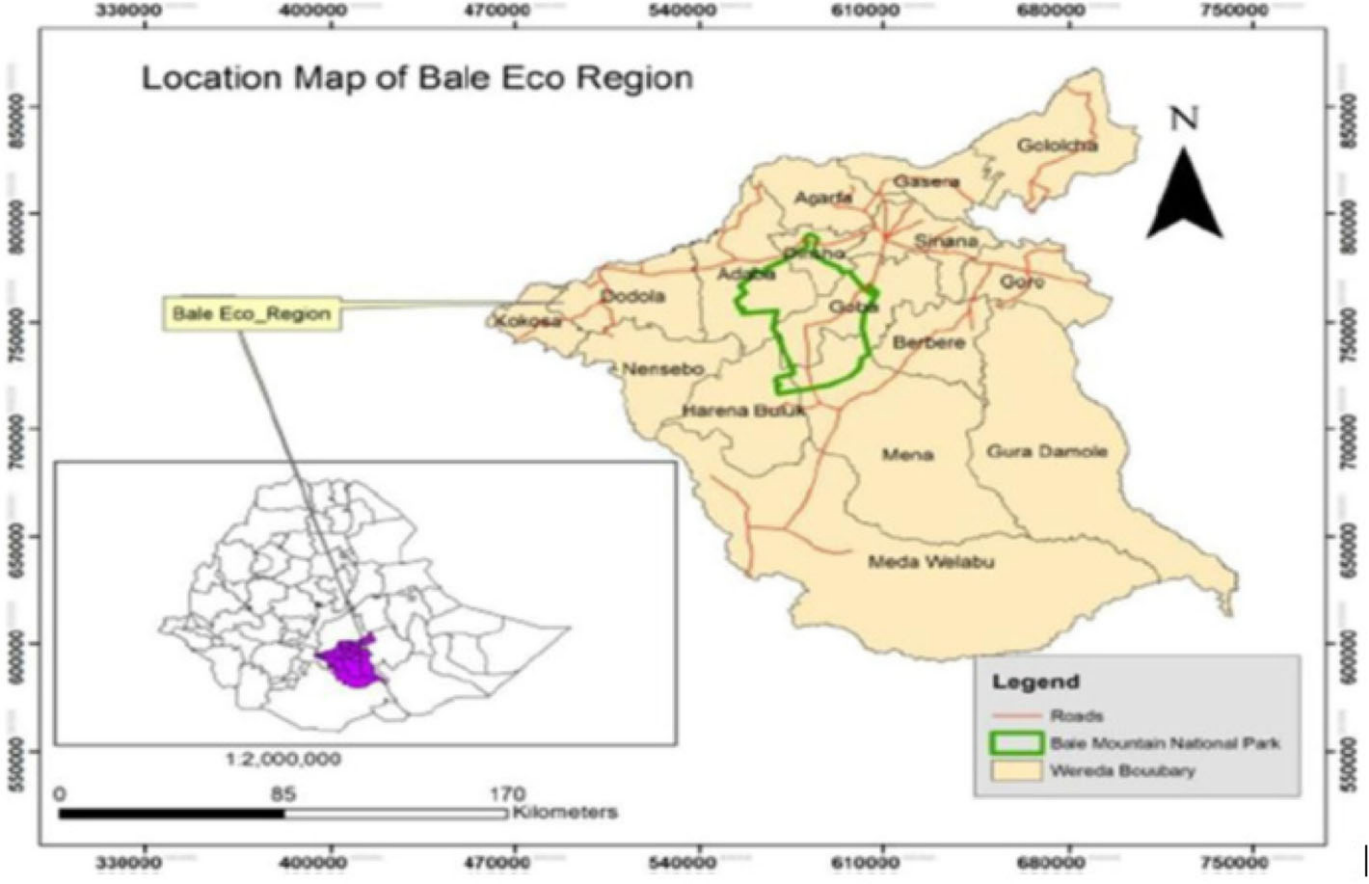
Locational map of Bale Eco-region and district boundaries, Southeast Ethiopia (Adopted from SHARE Bale Eco-region project document)

Out of sixteen districts of the Eco-region, twelve districts are located in Bale zone and four districts are located in West Arsi zone (Fig. 1). This study was conducted in six districts of Bale Eco-region (Dello-Mena, Harena-Buluk, Madda-Walabu, Goba, Dinsho, Berbere). The main town, “Bidre” of Madda Walabu district is the farthest and located at 626-k meter in the southeast direction from Addis Ababa (capital city of Ethiopia). There are 3 hospitals (Goba referral hospital, Dello-Menna hospital and Madda-walabu hospital (new partially functional)), 54 health centers, and 221 health posts in the study area and all are owned by the government. Family planning, antenatal care and delivery services are provided free of charge in all government health facilities located in the study districts. Modern contraceptive methods (injectable, pills, implants, male condoms) are available in all health facilities including health posts. Intrauterine contraceptive devices are available in health centers and hospitals, and permanent contraceptive method (surgical sterilization) is available in hospitals (unpublished *Bale zone health Office report, 2017*).

### Study population

The study population consisted of married couples who lived for more than six months in Bale Eco-region. Women who had at least one child were included in the study. However, those who were unable to respond or very sick were excluded.

### Sample size and sampling technique

The sample size for the household survey was determined by using single population proportion formula with the following assumptions: the proportion of maximum sample size to be 50%, with 95% confidence interval (CI) which is 1.96, and margin of error to be 4%. Adding non-response rate of 10%, the total sample size of 660 households were selected. Twenty-four focus group discussions (FGDs) consisting of married couples, and religious leaders were arranged. Similarly, 36 key informant interviews (KIIs) were arranged based on the need of the study.

For household survey, a multistage sampling technique was employed. Study districts were stratified into three Agro-ecologies; Highland (Dega), Midland (Weyina dega) and lowland (Kolla). From each stratum (Agro-ecology), two districts were selected by lottery method (Barbare and Madawalabu districts from lowland, Harena-Buluk, and Dello-Menna districts from Midland, Goba and Dinsho districts from highland). In the second sampling unit, 12 sub-districts (kebeles) (two kebeles from each district) were selected randomly. At kebele level, family folders (list of households) were used as the sampling frame and the required numbers of households were selected using systematic random sampling technique. In households with more than one eligible married women, one was selected by lottery method; however, if data collectors couldn’t find any eligible woman in a household, they shifted to the next immediate household.

For qualitative data, FGD participants were selected purposively based on who could give the most and best information about communities’ contraceptive practice. From each selected kebele, 4 FGDs were arranged; 2 groups consisted of married women and 2 groups consisted of married men. Each FGD consisted of 7 to 11 participants. *Gote development group leader*s were recruited for a key informant interviews. FGD participants and key informants were selected from each kebele with the help of health extension workers.

### Data collection

Pre-tested and structured questionnaire was used to collect quantitative data. The questionnaire was designed in English and translated into Afaan Oromo (a local language). The questioner was pre-tested outside the study area and some modification was made to have the final version. Trained enumerators who had the experience of data collection gathered the data through face-to-face interview. Twelve male and twelve female data collectors participated in the study. Four supervisors supervised the data collection process in each day.

For qualitative data, open-ended guides were prepared in English and then translated into the local language to explore perceptions of modern contraceptive methods. FGDs and key informant interviews were conducted with local language until no new findings emerged. The group discussions were moderated by university experts who speak the local language. Additional training on family planning and qualitative methods of data collection were also provided to the moderators. Study participants were given a subsequent code number based on their site (kebele, group and respondent number). During the discussion, each participant called the code number. In addition to audio recording, written notes were taken from the discussion.

### Measurements

Most of the questioners were taken from Ethiopian demographic health survey 2016 and other literatures were included to measure utilization of family planning methods [13–15, 18]. Furthermore, to explore the barriers to family planning utilization men and women were asked whether they had misconceptions regarding utilization of family planning methods. The guiding questions were: Would you tell us reasons for not using family planning methods? Yet again, participants who point out the reasons for not using family planning methods such as husband’s opposition, religious beliefs, concern and fear of side effects, and distance of family planning service, what do you know about it and other misconceptions were pursued. Probing questions were used to find out the phenomena of couple’s contraceptive practices within the community.

### Data analysis

Data were checked for completeness and inconsistencies. Epi-data version 3.1 was used for data entry and data were exported to SPSS version 21. Descriptive statistics were computed. Logistic regression analysis was used to identify the relationship between dependent and independent variables. Those independent variables which were significant in bivariate analysis (*P*-value < 0.05) were entered into the multivariable analysis. In the final model, a significant association was declared at a *p*-value less than 0.05. And finally, the results were presented in texts and tables with adjusted odds ratio (AOR) and the corresponding 95% confidence interval.

In qualitative data, all FGDs and KIIs were recorded and transcribed verbatim in Afaan Oromo. The typed narratives were then translated into English and verified for accuracy. No computer software was used for qualitative data analysis. Using thematic content analyses, the researchers read and reread the transcripts to be familiar with the data and a set of codes were developed to describe groups of categories with similar meanings. The grouped categories were refined and themes were generated from the text data manually. Direct quotations of FGD participants and KIIs were presented in italics to highlight key findings.

### Ethical considerations

Ethical clearance was obtained from research review committee of Madda Walabu University. Letters were secured from Bale zone health Bureau, Bale zone administrative and the respective districts of Bale Eco-region. Verbal informed consent was obtained from the study participants. All the information obtained from the study participants were kept confidential throughout the process of study, and the name of the participant was replaced by code. Withdrawal from the study at any point if they wished was assured.

## Results

### Socio demographic characteristics

A total of five hundred sixty-seven married women were successfully interviewed giving a response rate of 85.9%. Non-response rate was high due to absence of respondents despite enumerators repeated attempt to interview them. The mean age of the respondents was 30.52 (±7.36 SD). The age of the youngest participant was 19 year. More than half percent of women (55.6%) were between the age group of 21–30 years. The mean age at first marriage was 17.27(±1.7 SD). Majorities (88.6%) of the study participants were Muslim and 5% were Orthodox Christians. More than two-third of married women (64.6%) did not attend formal education while (18%) of women completed primary education. Regarding the household income, 53% and 41% of respondents had an annual income of 441–2200 and 22–440 dollars, respectively.

The average number of children a woman had was 4.7 (±1.6 SD), but 24.6% of women had more than seven deliveries. Two hundred thirty-three (41.1%) of women had more than seven pregnancies in the eco-region. Similarly, 57.3% of women spaced the last two children less than 24 months. For 47.7% of participants, their current pregnancy was unplanned. Eight percent (8.5%) of the participants reported that they had ever lost a child less than 5 years old. The mean number of future pregnancy desire among women was 4.8 (±1.3 SD). In this study, the mean length of postpartum abstinence and the mean duration of postpartum amenorrhea was 2.38 (±1.3 SD) and 8.61 (±3.8 SD) months, respectively. The mean number of postpartum insusceptibility to pregnancy was 9.7 (±1.3 SD) (Table 1).

**Table 1:** Socio-demographic and obstetric characteristics of women in Bale Eco-region, Ethiopia, 2017

Characteristics	Categories	N	%
Age of mother	< 20	29	5.1
	21–30	315	55.6
	31–40	164	28.9
	41–49	59	10.4
Mean age at first marriage	17.27 (±1.7 SD)		
Religions	Muslim	503	88.6
	Orthodox	36	6.4
	Protestant	19	3.4
	Catholic	9	1.6
Income in United States Dollar(USD)	22–440	233	41.1
	441–2200	305	53.8
	> 2200	29	5.1
Educational level of women	No formal education	366	64.6
	Primary education	104	18.4
	Secondary education	76	13.3
	College education	21	3.7
Parity	≤3	183	32.4
	4–6	246	43.6
	≥7	138	24.6
Gravida	≤3	157	29.5
	4–6	176	29.3
	≥7	233	41.1
Status of current pregnancy (*n* = 197)	Planned pregnancy	107	54.3
	Unplanned pregnancy	90	47.7
Birth interval between the last two children	≤24 months	324	57.3
	> 24 months	243	42.7
Mean duration of postpartum insusceptibility	9.71(± 1.12 SD) months		
How long you stayed to see monthly period after last delivery?	8.61(±3.87 SD) months		
How long you abstain from sex after year last delivery?	2.38(±1.13 SD) months		
Mean number of future pregnancy desire	4.8 (±1.3 SD)		
Ever had a child who died < 5 years old		48	8.5

### Family planning utilization

**Fig. 2:**
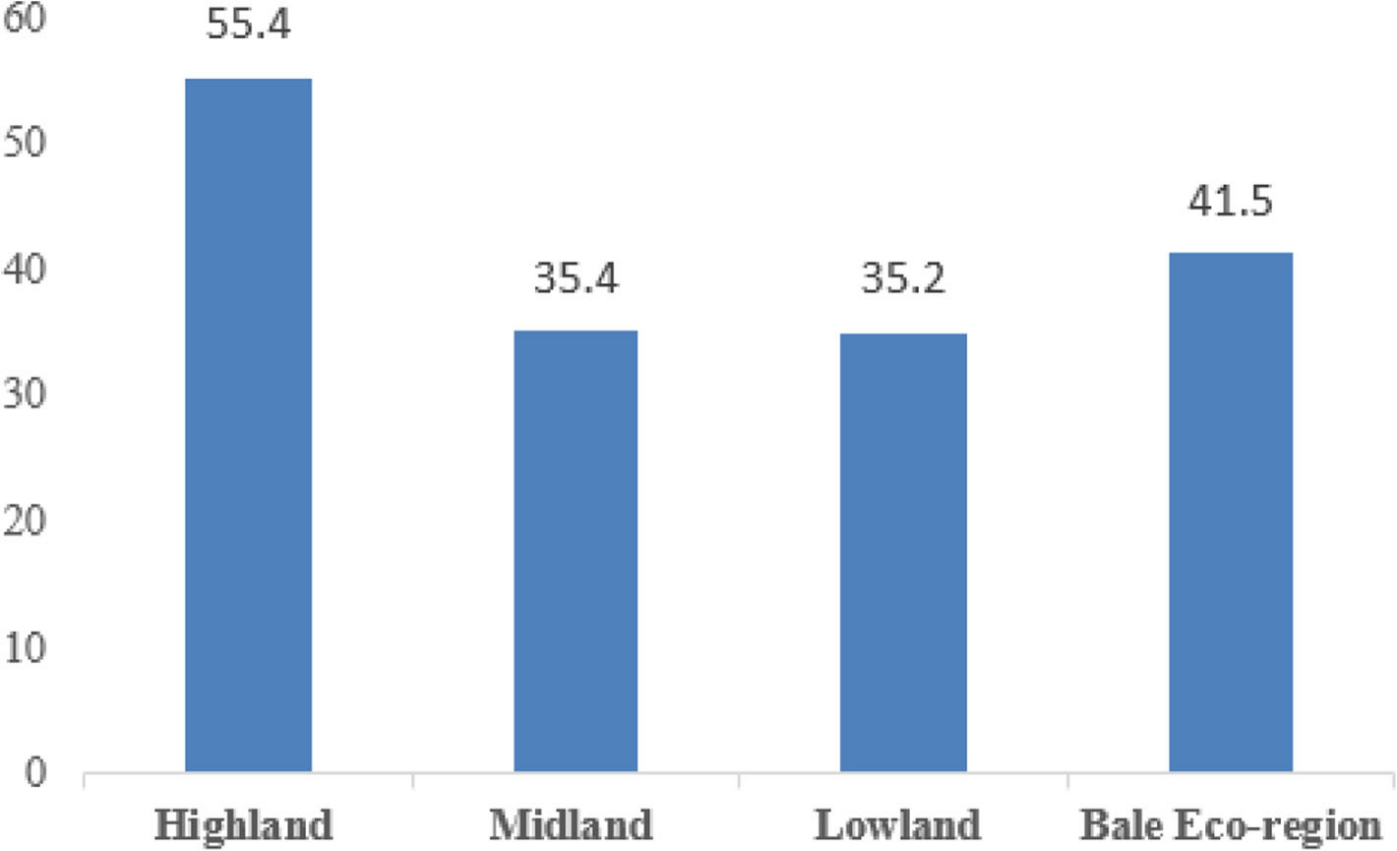
Frequency distribution of Modern contraceptive utilization among married women in Bale Eco-region, Southeast Ethiopia, 2017

**Fig. 3:**
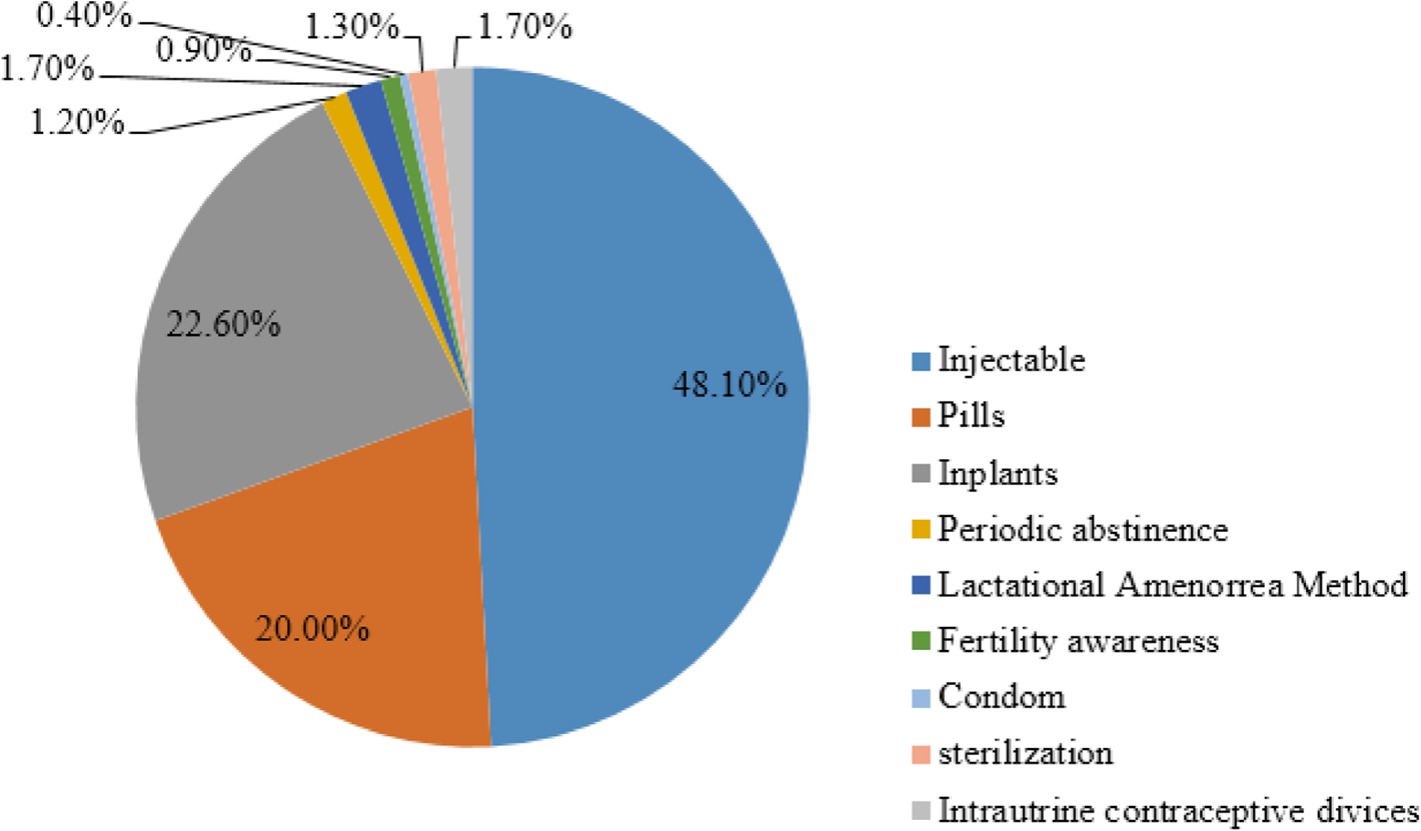
Family planning method mix among married women in Bale Eco-region, Southeast Ethiopia, 2017

**Fig. 4:**
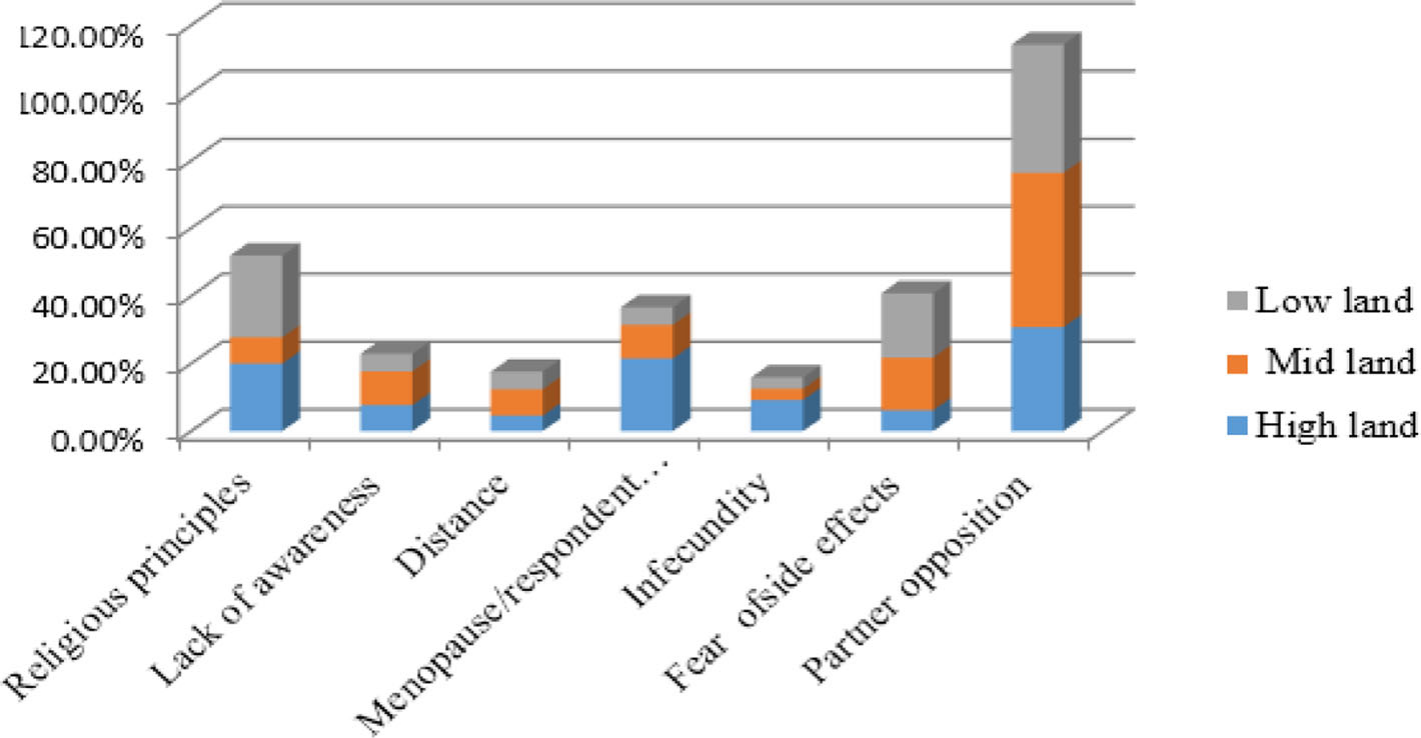
Reasons for not using modern contraceptive methods among married women in Bale Eco-region, Southeast Ethiopia, 2017

In this study, 41.5% of study participants were using modern contraceptive methods. Of the total contraceptive users, 55.4%, 35.4%, and 35.2% of women were from highland, midland and lowland residents, respectively (Fig. 2). This study also revealed that modern family planning method mixes; injectable (48.1%), implants (22.6%) and pills (20.0%) (Fig. 3). Those married women who were not using any modern contraceptive method during the survey were asked about the reasons for not using the methods. Accordingly, partner (husband’s) opposition (38.8%), religious principles (17.7%), concern and perceived fear of side effects (14.8%), long distance of FP service centers from home (5.9%), lack of awareness about contraceptive methods and services (7.4%), and health related problems (4.8%) were reported as a reasons for not using modern contraceptive methods (Fig. 4).

This study also employed qualitative data to triangulate findings of the household survey. FGDs and KII highlighted perceptions of married men and women for not using modern contraceptives in Bale Eco-region. Findings were described below with quotations for each theme.

### Male partner (husband’s) opposition

For some women, an opposition of modern family planning methods by their husbands or partners was a reason for not to use modern contraception despite their desire to do so. The main reasons for opposition include the desire for more children than the women herself wants, and believes that the use of FP goes against religious beliefs. One female FGD participant from Harena kebele (Group twelve respondent three (G12 R 3)) said that:

“*Most of the women in our ‘Gote’(sub-kebele) are not using modern FP because the husband proscribes them not to use family planning and get the services, husbands want another baby. But females are not ready”.*

Another male participant from Harena kebele (G11 R 7) said that:

*“My neighbor did not allow his wife to use FP; he concealed service appointment of his wife. He wanted his wife to keep having more children”.*

Another participant who failed to get FP methods reported that:

*“My husband strongly disagreed with me on family planning utilization. She added… he didn’t want me to get family planning service and he wants many children…he believes that children are wealth and given from God”.* Fasil-Sora kebele (G1 R 5).

In addition, KII indicated that male partners are responsible for women not using contraceptives.

*“Females did not use modern contraceptive methods without their husband’s approval because she has no power to decide contraceptive use. Some women go for FP services without the knowledge of their husbands since husbands might oppose their use of FP methods. Males want to have more children; some men say that modern contraceptive leads to illness”.* A female key informant from Wolti-kubsa kebele (KI-7).

### Concern and perceived fear of side effects

Across the three Eco- regions, fears of contraceptives side effects were reported as the reason for not using modern contraceptive; a female participant from Burkitu kebele (G16 R7) said that:

***“****Contraceptives had side effects;* women *do not have their monthly period while they are using injection and they stopped as a result of it”.*

Another participant also reported that misconstructions about modern contraceptives*.*

*“Use of modern contraceptives need sufficient food, but women didn’t get this enough food and then they became thin/malnourished”. Ware kebele (G17 R9).*

*“I heard implant result in infertility to get pregnant later on”.* Horosoba kebele (G 5 R9).

Likewise one female key informant from Miyo kebele reported that her concerns about the side effects of modern contraceptives.

*“I used implants and it resulted in excessive menses so I changed to depo” (KII 6).*

### Distance of health facility from home

Other reason for not using modern contraceptive was distance between communities and health facilities. An elder male participant from Oda Dima kebele *(G12 R 4)* said that:

*“Women were traveling on average 1.5 to 4 hours in order to get family planning methods and health care services: for example, a woman needs to travel for about four hours from Chiilankoo (zone 3) to Sefera (zone 1) in order to get contraceptive methods or service. Hence, family planning services should be available near to family planning users”.*

### Absence of husband from family planning sensitization training

FGDs also reflected that men had opposed FP use by their wife because they were not involved in FP information education communication session or advocating of contraceptive use in the community. One male participant (G20 R 7) reported that;

*“The FP training was given only for wife (male partners were not involved in family planning training). In addition, this FP sensitization training is convincing women and they are not clear about importance and utilization of family planning very well. Therefore, FP programs should not only target women”*. Haro-nano kebele (G 20 R7).

Another key informant from Delomena kebele reported that:

*“Male partner prohibits his wife to use family planning…..even he does not let her participate in community meeting as he has no idea at all and not informed about the benefits of FP training, and females are dependent on male decisions”. Delomena kebele (KII 9).*

### Religious principles and beliefs

The FGDs also brought to light the beliefs that the use of FP goes against certain religious beliefs. A male participant from Barisa kebele (G9 R 7) said,

*“We didn’t use family planning because of our religion; the Prophet had been said that one should marry a woman whose family has more children. Bearing many children is encouraged by Sherian Law”.*

Similarly, a Muslim female participant (G9 R 2) reported that:

*“Our religion prohibits it because the prophet said that the more children a woman bear the more the followers will grow”.*

Logistic regression analyses were performed between dependent (modern contraceptive use) and independent variables at (*P* < 0.05) level of significance. Multivariable analysis revealed that use of modern contraception among married women was associated with having more than seven deliveries (AOR = 2.98, CI = 1.91–6.10, *P* = 0.000) and having birth interval less than 24 months between the last two children (AOR = 3.8, CI = 13.41–21.61, *P* = 0.003).

## Discussion

In this study, on average, women had 4.7 live children and the mean number of future pregnancy desire among women was 4.8 (±1.3 SD). This finding was comparable with the national demographic health survey report [15] and study conducted in Jimma zone [20]. The mean length of postpartum abstinence and mean duration of postpartum amenorrhea were 2.38(±1.3 SD) and 8.61(±3.8 SD) months, respectively. The median number of postpartum insusceptibility to pregnancy was 9.7(±1.3 SD). This figure was slightly lower than the national figures where the median length of postpartum abstinence and postpartum amenorrhea among rural women were 2.5 & 10.9 months, respectively [21]. This supports the argument that as the household wealth increases, the median duration of amenorrhea, abstinence, and insusceptibility decline [15, 21].

The prevalence of modern contraceptive among study participants (41.5%) was higher than the studies in Hadiya zone (23.9%) [16], Afar region (8.5%) [12], pastoralist women (20.8%) in Bale eco-region [17] and comparable with a study in Jimma (43%) [20] and Illubabor zone (44.9%) [19]. It was also lower than a study reported in South Gondar (66.2%) [18], Gedeo zone (64.2%) [13]. This contraceptive prevalence rate variation could be the difference in access to health facilities and FP commodities in a study setting or it could be due to the intervention and awareness creation made by the government and non-governmental organizations on family planning services. Regarding the contraceptive method mixes, injectable (48.1%), implants (22.6%), and pills (20.0%) were the most contraceptive methods utilized by study participants. These findings were consistence with the Ethiopian health survey report [15] and a study in Jimma zone [20]. Eshete A and Belda et al. also reported that injectable, oral contraceptive pills, and implants were the main methods utilized [13, 17]. However, the most effective and long acting contraceptive method like intrauterine contraceptive device was the least common contraceptive, suggesting that health facilities in our study area were not able to deliver the service or acceptability of the aforementioned contraceptive method by the community was limited.

The current study identified some reasons for not using modern contraceptive methods. The most common reasons were spousal (husband’s) opposition (38.8%), religious principles (17.7%), concern and fear of side effects (14.8%), long distance of FP service (5.9%). Similar reasons were reported in another studies. Religious opposition (55.9%), fear of side effects (25.5%), and husband’s opposition (17.5%) were the reasons for non-use of modern contraceptive methods [17]. In Pakistan perceived side effects such as excessive bleeding and abdominal pain, infertility were the major reasons for not using contraceptive methods [8]. In Malawi, women discontinued modern contraceptives because of their perceived side effects and partner opposition [22]. Moreover, evidence from Nigeria [10] and Uganda [23] revealed that husband opposition was the major reason for not using modern contraceptives. Therefore, male’s forums on FP services should be arranged to make them involved in FP and to increase their responsibility for family planning.

Studies from Malawi [22] and Nkwanta district of Ghana revealed that distance of health facilities influenced modern contraceptives use [24]. Likewise, in Pakistani, the most common reason for not using modern contraceptive was lack of access family planning services [25]. The number of family planning units (health centers and health posts) should be increased to reach communities that were distant from the current FP services or home-based visit to family planning services should be initiated by health extension workers. Our qualitative study also assured that concern and fear of side effects, absence of males in FP information education communication sessions, and husband opposition were the reasons for not using contraceptives which were also reported in southern-Ethiopia and Kenya [9, 14, 26].

In the multivariate analysis, the number of children greater than seven and birth interval less than 24 months between the last two children were associated with current contraceptive use. Women having more than seven children were 2 times more likely to use contraceptives than those who had less than 4 children. This was in line with a study in Jimma zone, Gedeo zone, southern-Ethiopia [13, 20, 26]. Women who had birth interval less than 24 months between the last two children were more likely to use contraceptives than their counter part which had been confirmed by study in India [27]. This could be explained that women were ready to postpone or had the interest to limit the number of their children if birth interval was less than 24 months between the last two children. Hence, women should be encouraged to use family planning methods so as to reduce maternal and child mortalities as pregnancies spaced less than two years apart were considered to be high risk.

## Conclusion

The current contraceptive prevalence rate was still low as compared to other different parts of the region. The low contraceptive prevalence rate was mainly attributed to spousal (husband’s) opposition, religious beliefs, concern and fear of contraceptive side effects, distance of family planning service from home, lack of awareness about the methods and services, and health related problems. The number of children greater than seven and birth interval less than 24 months between the last two children were associated with the current contraceptive utilization. Therefore, continuous information education communication session opportunities about FP should be arranged to make male’s involved in FP services and to increase their responsibility for family planning use. Women should space their pregnancies by using family planning method as birth intervals less than 24 months were considered to be high risk for women and children. Due attention should be given to enhancing information, education and communication activities to correct concerns and fears around FP use and services. As a limitation, this study might be affected by interviewer bias and may overestimate the utilization of modern contraceptives. In addition, the risk for pregnancy or sexual activity was not assessed which could be a reason for non-use of contraceptives.

## Abbreviations

AOR: Adjusted Odds Ratio
CI: Confidence Interval
FGD: Focus Group Discussion
FP: Family Planning
KII: Key Informant Interview
SD: Standard Deviation
